# CO_2_ Permeability of Biological Membranes and Role of CO_2_ Channels

**DOI:** 10.3390/membranes7040061

**Published:** 2017-10-24

**Authors:** Volker Endeward, Mariela Arias-Hidalgo, Samer Al-Samir, Gerolf Gros

**Affiliations:** Molekular-und Zellphysiologie, AG Vegetative Physiologie–4220–Medizinische Hochschule Hannover, 30625 Hannover, Germany; Endeward.Volker@MH-Hannover.de (V.E.); Arias.Mariela@mh-hannover.de (M.A.-H.); Al-Samir.Samer@MH-Hannover.de (S.A.-S.)

**Keywords:** CO_2_ permeability, membrane cholesterol, protein CO_2_ channels, aquaporins, Rhesus proteins, aquaporin-1-deficient mice

## Abstract

We summarize here, mainly for mammalian systems, the present knowledge of (a) the membrane CO_2_ permeabilities in various tissues; (b) the physiological significance of the value of the CO_2_ permeability; (c) the mechanisms by which membrane CO_2_ permeability is modulated; (d) the role of the intracellular diffusivity of CO_2_ for the quantitative significance of cell membrane CO_2_ permeability; (e) the available evidence for the existence of CO_2_ channels in mammalian and artificial systems, with a brief view on CO_2_ channels in fishes and plants; and, (f) the possible significance of CO_2_ channels in mammalian systems.

## 1. Introduction

This review intends to update the state of this field as it has been given by Endeward et al. [1] in 2014. In addition, we attempt to give a compilation of all of the lines of evidence that have so far been published demonstrating the existence of protein CO_2_ channels and their contributions to membrane CO_2_ permeability. We also give a compilation of the recently described remarkable variability of the CO_2_ permeability in mammalian cell membranes. Because a discussion of the true value of intracellular CO_2_ diffusivity has come up [2], we estimate the relative roles of the intracellular diffusion resistance vs. the diffusion resistance of the cell membrane for exit of CO_2_ from the cell, and discuss the crucial role of the intracellular diffusion coefficient for this relation. A novel development in the field is the prominent role, which membrane cholesterol seems to play in determining the CO_2_ permeability of a large number of mammalian biological membranes [3]. A further, newly evolving principle is the amazingly good positive correlation between the values of cellular oxygen consumption and CO_2_ permeability, suggesting that cells adapt their CO_2_ permeability to their level of CO_2_ production. Finally, we extensively discuss the role of CO_2_ channels for gas exchange by red cells, since this is presently the in vivo system whose CO_2_ transport properties have been characterized most quantitatively and in the most detail [4,5,6]. 

## 2. Measurement and Variability of CO_2_ Permeabilities of Biological Membranes

Table 1 compiles the CO_2_ permeabilities that we have measured with the ^18^O mass spectrometric method for a broad variety of cells or organelles. Derivation of P_CO2_ from mass spectrometric recordings of the time course of C^18^O^16^O in cell suspensions requires an extensive system of mathematical equations describing the complete process of ^18^O exchange between CO_2_, HCO_3_^−^, and H_2_O. However, it nevertheless constitutes at present the most direct method to obtain quantitative estimates of cell membrane permeabilities. The method has been introduced in a simpler form without consideration of the CO_2_ permeability by Itada and Forster [7]. The theory, as developed further by our group, as well as the handling of the technique, have been presented in their definitive form by Endeward and Gros [8], and several strengths and limitations of the method have been extensively discussed by Endeward et al. [1]. Very recently, another group has used this ^18^O technique to measure the CO_2_ permeability of isolated chloroplasts [9]. Unfortunately, the authors were not able to measure the internal stroma carbonic anhydrase activity of the chloroplasts, a quantity that is essential for the evaluation of the mass spectrometric records [8]. Lack of precise knowledge of this figure is expected to constitute a limitation to the reliability of the derived CO_2_ permeabilities.

Another approach that is often used to estimate membrane CO_2_ permeabilities, is that employed by Boron’s group [4,10,11], which records the alkaline pH shift on the cell surface that is associated with an influx of CO_2_ into oocytes. This allows one to indirectly quantitate that flux. It is clear that the surface pH amplitude is related to membrane CO_2_ permeability, but it is hardly feasible to derive an explicit value of P_CO2_ from these pH shifts [12,13]. So the method can be considered semi-quantitative, but has been important in providing further corroboration of the gas channel property of aquaporin 1 and has successfully been used to provide the important comparison of the CO_2_ permeabilities of the aquaporin and Rhesus protein isoforms [10,11,14].

A further important method to quantitate membrane CO_2_ permeabilities, especially of artificial phospholipid vesicles, is the stopped-flow technique. An intravesicular fluorescent pH indicator is used to follow the uptake of CO_2_ by the vesicles. This requires extremely rapid intravesicular conversion of CO_2_ into HCO_3_^−^ and H^+^, i.e., the presence of high activities of carbonic anhydrase inside the liposomes. Tsiavaliaris et al. [15] have shown that in some earlier studies this approach has suffered from (a) an intravesicular carbonic anhydrase activity that was not sufficient to render the CO_2_ hydration reaction non-limiting, in combination with (b) the use of an equation describing the process of CO_2_ uptake that assumes instantaneous chemical equilibrium of CO_2_, HCO_3_^−^, and H^+^. This led to erroneously low P_CO2_ values for liposomes of around 0.001 cm/s [16,17]. When these problems are circumvented, it can be shown that cholesterol-free liposomes have a P_CO2_ of >0.1 cm/s, and liposomes with 50% cholesterol have a P_CO2_ of 0.01 cm/s [15]. As will be discussed below, these P_CO2_ values agree very well with those obtained with the mass spectrometric ^18^O technique. Thus, the stopped flow method seems very suitable in principle to quantitate CO_2_ permeabilities. However, because of the high pressures in the syringes and the high flow rates arising in the mixing chamber, it will be difficult to apply this technique to cell suspensions when cell lysis is to be avoided. 

Table 1 shows the CO_2_ permeabilities determined by our lab for various cellular and organellar membranes, all by the ^18^O technique. The P_CO2_ values vary enormously, by a factor of 200, from 0.0015 to 0.3 cm/s. This seems to suggest that P_CO2_ is a highly regulated quantity and helps cells adapt to physiological challenges, whose nature shall be discussed below.

## 3. Is There a Physiological Parameter Governing the Adaptation of CO_2_ Permeabilities?

**Figure 1:**
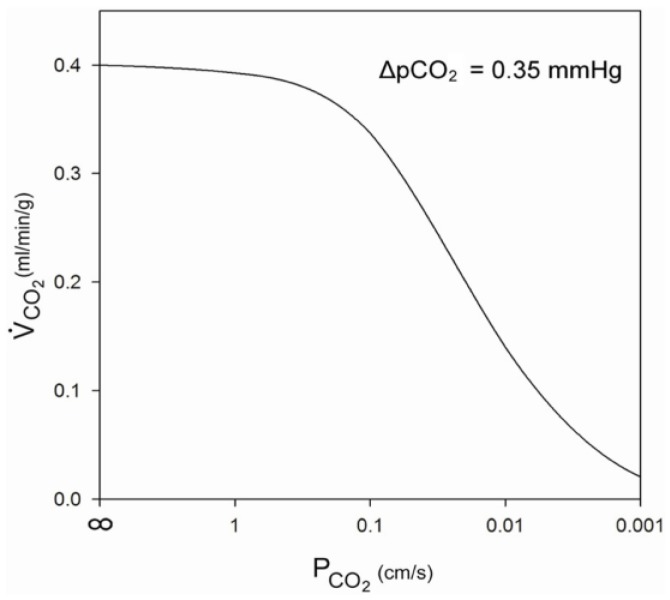
Influence of membrane CO_2_ permeability on CO_2_ release from a maximally metabolizing cardiomyocyte at a given partial pressure difference of 0.35 mmHg over the half-thickness of the cell. From: Endeward et al. [1].

We ask here, whether the variability of the CO_2_ permeabilities seen in Table 1 serves a definable physiological purpose. Endeward et al. [1] have theoretically shown that the resistance of the cell membrane can become significant for cellular CO_2_ release—in addition to that of the cytoplasmic diffusion path—when P_CO2_ falls below about 0.1 cm/s. They also showed that, for a given intra- to extracellular CO_2_ partial pressure gradient, a membrane P_CO2_ of, say, 0.01 cm/s would markedly limit CO_2_ exchange of a cell of very high oxidative metabolic rate, such the 300 nmol O_2_/g tissue/s (=0.40 mL O_2_/g tissue/min) found in maximally working cardiomyocytes [20], and reduce CO_2_ efflux to about 1/4 of what is required (Figure 1). However, for a cell of a low O_2_ consumption, such as exhibited by cells in culture, e.g., with the number of 12 nmol O_2_/g cell/s (0.016 mL O_2_/g tissue/min) reported for MDCK cells [21], this same P_CO2_ would have no effect on the rate of CO_2_ exchange at all. This can also be appreciated from Figure 1. We should note that a limitation of CO_2_ efflux by a high membrane diffusion resistance would require a build-up of the intracellular CO_2_ partial pressure to ensure sufficient CO_2_ removal. This would imply an increased acid load for this cell. 

In view of these considerations, it appears conceivable that the value of membrane CO_2_ permeability is adapted to the level of aerobic metabolism of the cell, i.e., to the level of the rate of CO_2_ production.

To test this hypothesis, we can take a look at the specific rates of oxygen consumption of some of the CO_2_ permeabilities of Table 1. As just mentioned, MDCK cells with a P_CO2_ of 0.017 cm/s have an oxygen consumption of 12 nmol O_2_/g cell/s, rat cardiomyocytes exhibiting a P_CO2_ of 0.1 cm/s possess an O_2_ consumption of 300 nmol O_2_/g tissue/s, and rat liver mitochondria with a P_CO2_ of 0.33 cm/s have a maximal respiratory rate of 1000 nmol O_2_/mL/s [19]. Thus, there is a clear increase of P_CO2_ with an increasing rate of O_2_ consumption or CO_2_ production, respectively. This principle will have to be further substantiated by obtaining more data pairs of P_CO2_ and O_2_ consumption for various tissue cells. However, we can already conclude from these values that P_CO2_ of cell membranes appears to be adapted to—and possibly regulated by—the level of oxygen consumption.

Other physiological processes to which membrane CO_2_ permeabilities seem to adapt in a major way are (a) the especially high rate of gas exchange across red blood cell membranes, and possibly (b) the high rate of CO_2_ diffusion across the apical membrane of the proximal tubule required for tubular bicarbonate reabsorption. Both these adaptations will be discussed below.

## 4. Cholesterol—One of Two Mechanisms Regulating CO_2_ Permeability

Two factors that affect the CO_2_ permeability of a membrane have been identified so far: the presence or absence of protein gas channels, and the content of cholesterol in the membrane. Gas channels have been expressed in oocytes to demonstrate this, and 90% of the CO_2_ permeability of the human red cell membrane has been shown be due to the channels aquaporin-1 and Rhesus-associated glycoprotein. This evidence for gas channels will be discussed in detail below.

**Figure 2:**
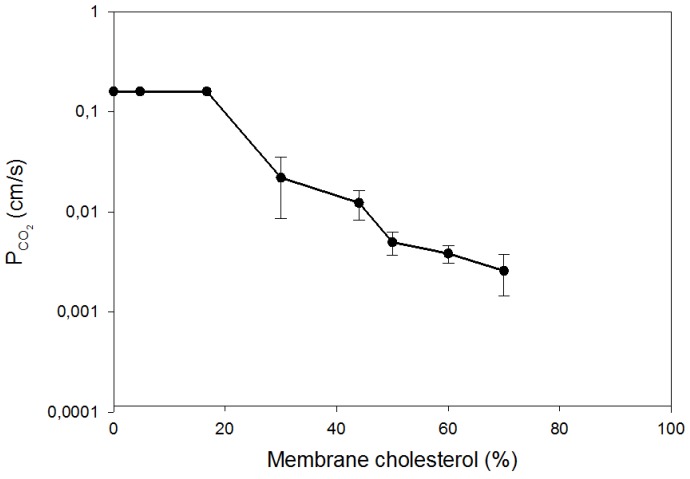
CO_2_ permeability (P_CO2_) of phospholipid vesicles with variable concentration of cholesterol as a function of cholesterol content in mol % cholesterol per mol total membrane lipids. P_CO2_ determination by the mass spectrometric ^18^O exchange method. From: Itel et al. [3].

The second important determinant of P_CO2_ is the content of cholesterol in the membrane. As shown in Figure 2, this has been studied systematically by Itel et al. [3], who used artificial phospholipid vesicles with cholesterol contents of between 0% and 70%, and measured their CO_2_ permeability by the ^18^O mass spectrometric method. Between 20% and 70% cholesterol P_CO2_ of these vesicles falls by two orders of magnitude, from ≥0.16 cm/s to about 0.002 cm/s. Thus, between these concentrations, P_CO2_ depends drastically on cholesterol. It is in agreement with other observations on cholesterol in membranes that there is no effect of cholesterol on P_CO2_ between 0% and 20% (c.f. [3]). 

**Figure 3:**
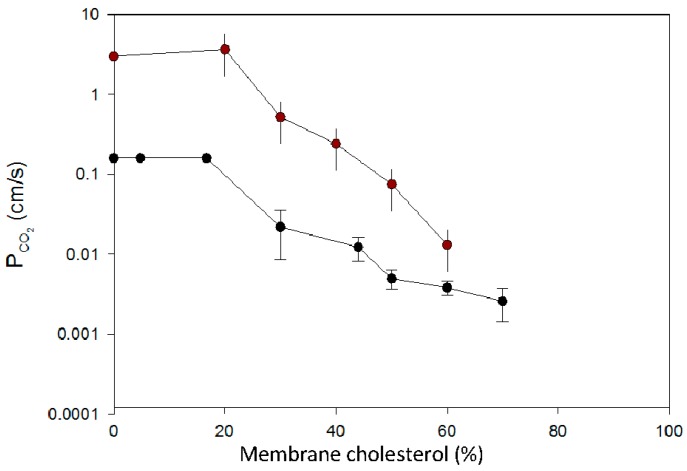
Cholesterol dependency of the CO_2_ permeability of phospholipid vesicles. Upper curve: calculated from the molecular dynamics simulation data of Hub et al. [22], lower curve: experimental curve of Itel et al. [3] as shown in Figure 2.

Figure 3 shows a dependency of P_CO2_ on membrane cholesterol as it can be derived from the molecular dynamics simulation of Hub et al. [22] (upper curve) in comparison to the experimental curve by Itel et al. [3] (lower curve). The starting points of P_CO2_ at 0% cholesterol are about an order of magnitude apart, possibly because Hub et al. based their calculation on a mixture of phospholipids quite different from the mixture used by Itel et al. However, in all other respects, there is an astonishing agreement between theory and experiment: (a) Hub’s curve as well as Itel’s curve fall by about two orders of magnitude between 20% and 60 to 70% cholesterol content in the membrane, and (b) Hub et al. [22] conform with the observation of Itel et al. [3] that cholesterol does not affect P_CO2_ between concentrations of 0 and 20%. Thus, molecular dynamics calculations and ^18^O measurements of liposomes both show the drastic reduction of P_CO2_ by increasing concentrations of cholesterol. 

Recently, for the case of CO_2_, this effect has been confirmed by an entirely different experimental technique, i.e., stopped flow observations of the uptake kinetics of CO_2_ by the type of vesicles studied by Itel et al. [3]. With this technique, Tsiavaliaris et al. [15] have observed a reduction of P_CO2_ of liposomes from >0.1 cm/s to 0.01 cm/s, when membrane cholesterol was increased from 0 to 50%. This effect agrees well with what one predicts from the curve of Itel et al. [3], as shown in Figure 2. Thus, three entirely different approaches lead to the same result: a most drastic effect of cholesterol on membrane CO_2_ permeability.

Another paper, by Kai and Kaldenhoff [23], uses pH measurements on the surface of planar lipid bilayers to measure lipid bilayer P_CO2_, a method introduced by Missner et al. [24]. They find a six times lower P_CO2_ in phosphatidylcholine bilayers with 67% as compared to 0% cholesterol. Thus, their measurements confirm a strong effect of cholesterol, but not as strong as that observed by Itel et al. [3], Hub et al. [22] and Tsiavaliaris et al. [15], who would predict an effect of around two orders of magnitude by 67% cholesterol. It should be noted that in an early study, Missner et al. [24] report no effect of cholesterol at all on P_CO2_ of phospholipid bilayers. We add that these latter discrepancies may be related to the difficulty of achieving the same ratio of cholesterol/total lipids in the membranes of the liposomes as established in the solution from which the liposomes are generated. In our experience, the final liposomes sometimes take up substantially less cholesterol than is present in the solution, although they never seem to take up more cholesterol/total lipids than present in that solution. 

The strong cholesterol effects on P_CO2_ observed by stopped flow, ^18^O exchange and molecular dynamics simulation, are paralleled by findings of Hill and Zeidel [25] of a 45-fold decrease in water permeability, when 52% cholesterol was added liposomes containing 9% phosphatidycholine, 18% sphingomyelin, and 21% glycosphingolipid. Similarly, the same maneuver reduced in their work the permeabilities for formamide by 54-fold, for acetamide by 130-fold, for urea by 20-fold, and for NH_3_ by 90-fold. Since CO_2_ is quite hydrophilic [26], it is especially interesting that both water and the hydrophilic NH_3_ exhibit a similar or greater reduction of their permeability by cholesterol when compared to CO_2_. We may add that Zocher et al. [27] have observed by molecular dynamics simulation a reduction of NH_3_ permeability in the presence of 50% cholesterol to 3% of its value in the absence of cholesterol, a reduction approaching that observed experimentally for NH_3_ by Hill and Zeidel [25], and similar to the reduction of P_CO2_ measured by Itel et al. [3] (Figure 2). 

**Figure 4:**
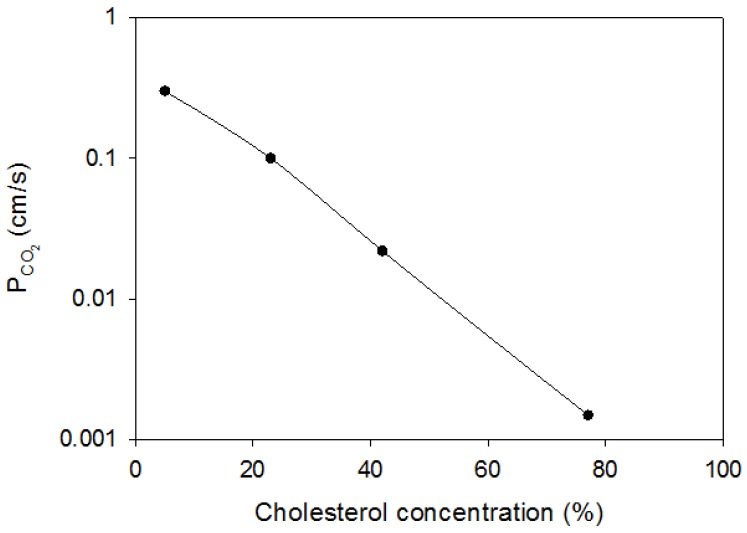
CO_2_ permeability of biological membranes as a function of their cholesterol content (% mol cholesterol/mol total membrane lipids). From left to right: rat liver mitochondria [17], cardiomyocytes [18], basolateral membrane of proximal guinea pig colon [8], and apical membrane of proximal guinea pig colon [8].

We conclude from this discussion that the available evidence predominantly suggests that cholesterol can reduce the CO_2_ permeability of membranes by about two orders of magnitude like it does with other hydrophilic (and non-hydrophilic) substrates. A further important support for this view comes from the measurements of the CO_2_ permeability of various biological membranes, which exhibit different concentrations of cholesterol but lack protein gas channels. Some such measurements have been reported by Itel et al. [3], and others are given above in Table 1. In Figure 4, we have plotted the P_CO2_ values of these biological membranes versus their cholesterol content as taken from the literature. The permeabilities in the figure are those for mitochondria [19], cardiomyocytes [18], the basolateral membrane of proximal colon epithelium [8], and the apical membrane of proximal colonic epithelium [8]. None of these membranes has a significant amount of protein CO_2_ channels. It is apparent in a semilogarithmic plot, like the one used in Figure 2 for liposomes, that there is an almost linear relation between P_CO2_ and cholesterol. Quite similar to what is shown in Figure 2 for liposomes, P_CO2_ falls by a little more than two orders of magnitude, when membrane cholesterol is raised from 5% to 77%. This shows that cholesterol is as powerful in lowering P_CO2_ in biological membranes as it is in artificial liposomes. 

## 5. Significance of Membrane vs. Cytoplasmic Diffusion Resistance

Figure 1 suggests that membrane CO_2_ permeability can affect cellular CO_2_ release when it falls below a value of 0.1 cm/s. This calculation is based on a certain intracellular cytoplasmic diffusion resistance R_cyto_, which is given by an assumed intracellular diffusion path length d from mitochondria to plasma membrane of 7 μm, and an intracellular CO_2_ diffusion coefficient D_CO2_ of 1.3 × 10^−5^ cm/s, using the Equation (1):

R_cyto_ = d/D_CO2_. (1)

The total cellular CO_2_ diffusion resistance is then given by the sum of cytoplasmic and membrane diffusion resistances, i.e.,

R_tot_ = R_cyto_ + R_membr_ = d/D_CO2_ + 1/P_CO2_. (2)

This equation illustrates that the membrane resistance R_membr_ becomes important when it contributes significantly to the total resistance. With the numbers given above, R_cyto_ is 54 s/cm, and with P = 0.1 cm/s, R_membr_ is 10 s/cm, i.e., the membrane contributes 15% of the total diffusion resistance. 

It is clear that the relative contribution of the membrane resistance depends on the value of the intracellular CO_2_ diffusion coefficient. Recently, it has been suggested that the intracellular CO_2_ diffusion—at least in the case of red blood cells—is much lower than thought heretofore [2]. For this reason, we will discuss the available evidence for the value of the intracellular diffusion coefficient of CO_2_. 

**Figure 5:**
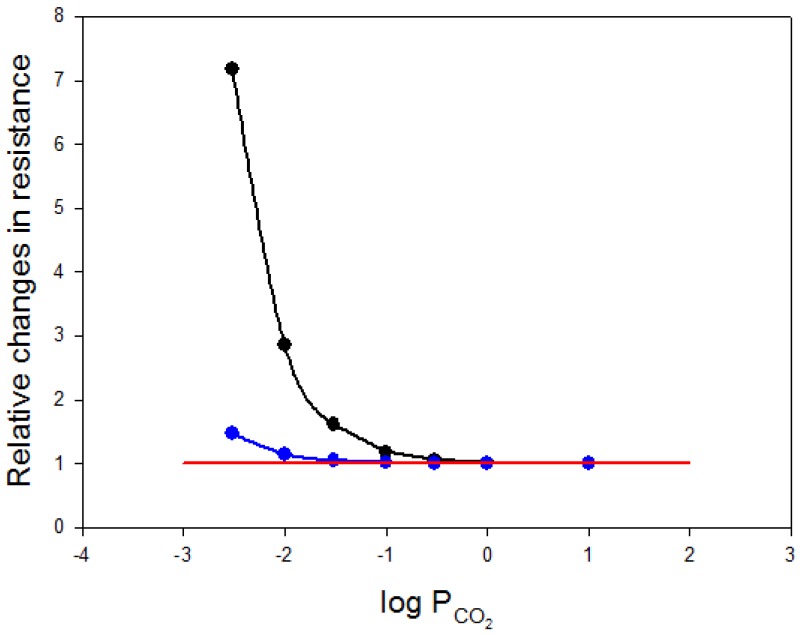
Relative changes in the combined diffusion resistances of 7 μm of cytoplasm and the cell membrane (*Y*-axis), versus varying values of CO_2_ permeability (*X*-axis). Relative changes in total resistance are defined as total resistance per resistance of the cytoplasm alone. Note that P_CO2_ on the *X*-axis is plotted logarithmically and varies between 10 and 0.003 cm/s. The upper curve (black) is calculated for an intracellular CO_2_ diffusion coefficient of 1.3 × 10^−5^ cm^2^/s, the lower curve (blue) for a more than 10 times lower D_CO2_ of 0.104 × 10^−5^ cm^2^/s as it has been postulated by Richardson and Swietach [2]. The horizontal red line represents total diffusion resistance for infinitely high P_CO2_, i.e., the normalized cytoplasmic resistance alone. It is apparent that, for “normal” P_CO2_ values between 0.02 and 0.3 cm/s (Table 1), the relative contribution of membrane CO_2_ permeability to the total cellular diffusion resistance becomes almost negligible for this very low cytoplasmic CO_2_ diffusivity (blue curve), whereas for the “classical” diffusion coefficient of 1.3 × 10^−5^ cm^2^/s (black curve) this contribution rises enormously with lower P_CO2_ values.

Richardson and Swietach [2] uncaged protons locally within red cells from 6-nitroveratraldehyde and observed the dissipation of these protons by imaging the intracellular fluorescence of a pH indicator. The kinetics of dissipation essentially reflects the facilitated transport of H^+^ by translational hemoglobin diffusion [28,29] and by the combined reaction and diffusion processes of H^+^, HCO_3_^−^, and CO_2_. The latter also involves, among other steps, the intraerythrocytic diffusion of CO_2_. If the complete process of facilitated H^+^ transport is modelled correctly, in principle, information on CO_2_ diffusivity can be derived from the fluorescence measurements. It is clear that the entire process is highly complex and further complicated by the red cell geometry, and that this approach to determining intraerythrocytic CO_2_ diffusion is quite indirect. In their article, Richardson and Swietach [2] come to the conclusion that the CO_2_ diffusivity inside red cells is 23 times lower than in water. With a D_CO2_ in water of 2.4 × 10^−5^ cm^2^/s (37 °C; [30]) R_cyto_ becomes 671 s/cm. This value of course renders a contribution of the membrane to R_tot_ of 10 s/cm entirely insignificant. This is illustrated in Figure 5, which shows the relative change in total resistance as a function of P_CO2_. The lower curve (blue) has been calculated for an intraerythrocytic CO_2_ diffusion coefficient of 2.4 × 10^−5^/23 = 0.10 × 10^−5^ cm^2^/s, as reported by Richardson and Swietach [2], the upper curve (black) represents the same calculation for the classically used D_CO2_ of the order of 1.3 × 10^−5^ cm^2^/s [31]. Whereas, the black curve shows a 7-fold increase in total resistance when P_CO2_ is lowered to an extreme 0.003 cm/s, total resistance increases by only 30% in the case of the blue curve for the low D_CO2_. Thus, whether low P_CO2_ values are relevant for cellular CO_2_ release, depends strongly on the true intracellular CO_2_ diffusion coefficient. 

What is the evidence for a much higher intraerythrocytic CO_2_ diffusion coefficient than that postulated by Richardson and Swietach [2]? Gros and Moll [31] have reported CO_2_ diffusion measurements across 1 mm thick layers of packed intact mammalian red cells, hemolysed red cells, and of pure hemoglobin solutions of identical hemoglobin concentration. In all three cases, they obtained practically identical D_CO2_ values of 1.14 × 10^−5^ cm^2^/s at 37 °C. Very similar results have been obtained for the diffusion of O_2_ across layers of red cells and hemoglobin solutions of identical hemoglobin concentration by Kreuzer and Yahr [32] and by Kutchai and Staub [33]. Although these results immediately seem incompatible with the idea of a 10 times lower “true” intracellular CO_2_ (or O_2_) diffusivity, one might ask whether in layers of packed red cells the extracellular, intercellular, fluid might constitute a shunt pathway of hemoglobin-free fluid, and consequently of low diffusion resistance. The volume fraction of extracellular fluid in centrifuged red cells has been estimated to be ~2% [34]. Gros and Moll [31] report a hemoglobin concentration of packed beef red cells of 33 g/100 mL, while the intraerythrocytic hemoglobin concentration is ~34 g/100 mL (Documenta Geigy Wissenschaftliche Tabellen [35]). This would imply an extracellular space of 3% in their packed red cells. A “shunt” pathway for CO_2_ through this extracellular space would be most effective under the unrealistic condition that the entire extracellular space constitutes a coherent parallel diffusion resistance of a path length identical to the thickness of the layer. The diffusion resistance of this composite layer can then be obtained as the sum of reciprocals of parallel resistances of (i) packed red cells, comprising 97–98% of the diffusion area, and of (ii) a water column comprising 2–3% of the diffusion area. Thus, with 1/R_tot_ = 1/R_ery_ + 1/R_water_, we can write (3)

1/R_tot_ = (0.98 × 1.14 × 10^−5^ cm^2^/s) + (0.02 × 2.4 × 10^−5^ cm^2^/s)  = 1.12 × 10^−5^ cm^2^/s + 4.8 × 10^−7^ cm^2^/s = 1.17 × 10^−5^ cm^2^/s, (3)

where the diffusion areas have been normalized and the thickness of the diffusion layer has been omitted, because it is identical for both components. Thus, for an extracellular space of 2%, the intracellular diffusivity of CO_2_ is overestimated by 2%, and for an extracellular space of 3%, it is overestimated by 3%. Conversely, with the above-mentioned low intracellular CO_2_ diffusion coefficient of 0.104 × 10^−5^ cm^2^/s inserted into equation (3), we predict an apparent D_CO2_ of 0.15 × 10^−5^ cm^2^/s for this experiment, almost 10 times lower than actually measured. In reality, the extracellular shunt diffusion paths will run around red cells and thus be much longer than the thickness of the layer, and this will even further reduce their contribution to 1/R_tot_ of the layer. Therefore, the measurements of gas diffusion through layers of packed cells will, for CO_2_ as well as for O_2_, lead to diffusion coefficients hardly different from the true intracellular gas diffusion coefficients. We conclude that a 10 times lower D_CO2_ inside red cells is clearly incompatible with these diffusion measurements. Thus, the role of membrane P_CO2_ will be as shown by the upper black curve of Figure 5, rather than as that illustrated by the lower blue curve. It is expected that the same conclusion holds for other tissues such as muscle [31]. By the same token, P_CO2_ will affect CO_2_ release by tissues in about the way illustrated in Figure 1. We may finally consider how the intraerythrocytic CO_2_ diffusion coefficient influences the time required by the blood in the lung capillaries to release its CO_2_ (see Table 2 below). As seen in the first line of Table 2, the process of RBC CO_2_ release in the absence of an effect of the cell membrane is estimated to be completed by 95% after 50 ms, when the classical intracellular CO_2_ diffusivity is used. With the 10 times lower CO_2_ diffusivity of Richardson and Swietach [2], this time is calculated to be prolonged to 480 ms, almost 10-fold. Such a time interval may exceed the capillary transit time available in the lung under conditions of heavy exercise.

## 6. Evidence for Protein CO_2_ Channels

The earliest evidence for the existence of membrane proteins that conduct CO_2_ came from (A) analyses of the CO_2_ permeability of red cells and (B) the observation of CO_2_ fluxes into aquaporin 1-expressing Xenopus laevis oocytes. 

(A) Forster et al. [36] observed in human red cells using the ^18^O-exchange technique that 4,4′-diisothiocyanato-stilbene-2′2′-disulfonate (DIDS) not only inhibits the permeation of HCO_3_^−^ by inhibiting the Cl^−^–HCO_3_^−^ exchanger, but in addition, affects the mass spectrometric signal in an additional way. By applying the extended theory of ^18^O-exchange developed in our lab [37], they were able to show that this additional effect constituted an inhibitory effect of DIDS on the membrane permeability for CO_2_. They concluded that the membrane must possess a protein that acts as a CO_2_ channel, and that this channel is inhibited by DIDS. These studies were followed by studies of human red cells lacking potential candidates for the role of a CO_2_ channel. Firstly, aquaporin-1-deficient human red cells (RBC) were shown by Endeward et al. [4] to exhibit (1) only about 50% of the CO_2_ permeability of normal RBCs, (2) a reduced but still present inhibitability by DIDS, together with (3), a largely lost inhibitability by pCMBS. The presently likely but not fully proven explanation for the behavior of these inhibitors is that one part of the aquaporin-1 (AQP1)-mediated CO_2_ flux uses the AQP1 water channel of the AQP1 monomer and is therefore inhibited by the inhibitor of this channel pCMBS. DIDS, which is not an inhibitor of the AQP1 water channel, is thought to inhibit the flux of CO_2_ that occurs through the central pore of the AQP1 tetramer [38]. Therefore, the effect of pCMBS is lost, when AQP1 is lacking. 

Secondly, Endeward et al. [5,6] studied human RBCs that were devoid of the Rhesus protein complex, including the Rhesus-associated glycoprotein RhAG, which had then already been known to provide a channel for NH_3_ [39]. Endeward et al. [5,6] found that RhAG-deficient RBC (1) possess only 50% of the CO_2_ permeability of normal RBC; (2) exhibit a reduced but still present inhibition by DIDS; and, (3) show a competition between the transport of CO_2_ and NH_3_, suggesting that CO_2_ and NH_3_ use the same channel in RhAG; (4) exhibit no effect of the more specific inhibitor of the Cl^−^–HCO_3_^−^ exchanger DiBAC on CO_2_ permeability. Since both AQP1 and Rh protein complex are blood groups in human RBC, the authors also studied several other blood group deficiencies, finding that none of the other blood group proteins acts as a CO_2_ channel. From all of these studies, it can be concluded that at least 90% of the CO_2_ permeability of the human red cell membrane is due to the two CO_2_ channels AQP1 and RhAG. Both of these channels are inhibited by DIDS, which makes DIDS a quite efficient gas channel inhibitor, in line with the original findings by Forster et al. [36].

While normal human RBC exhibit a CO_2_ permeability of 0.15 cm/s, AQP1-deficient RBC in the presence of 100 μM DIDS have a P_CO2_ of 0.015 cm/s only. Human red cells possess a cholesterol content in their membrane of 45% [40,41]. Inspection of Figure 2 as well as Figure 4 shows that about a value of 0.015 cm/s for P_CO2_ can be expected for a membrane cholesterol of 45%. This low P_CO2_ can be considered the “intrinsic” P_CO2_ of this membrane. The actual value is ten times higher due to the presence of two protein CO_2_ channels that occur in this membrane in large numbers [42]. The CO_2_ permeability of several other cells, such as those shown in Figure 4, is exclusively defined by their membrane’s cholesterol content. The red cell, by contrast, has a comparatively high cholesterol content, but, presumably on account of its central role in gas transport, compensates the effect of this on P_CO2_ by the incorporation of two abundant gas channels. It should be noted that apparently a majority of cells regulate their CO_2_ permeability by their membrane’s cholesterol content, only a lesser number of cells use gas channels in addition. 

(B) Further important evidence for the existence of gas channels comes from work on AQP1-expressing oocytes. Nakhoul et al. [43] and Cooper and Boron [44] showed by intracellular pH recordings that the influx of CO_2_ into oocytes is accelerated by about a factor of two upon expression of AQP1. Later on, this group used the more sensitive surface pH measurements to follow the influx of CO_2_ into oocytes. Endeward et al. [4] showed that after the expression of AQP1 in oocytes the alkaline surface pH transient associated with CO_2_ influx was drastically enhanced and was inhibitable by DIDS. Musa-Aziz et al. [10] also used surface pH measurements on oocytes expressing the particular proteins, to confirm the previous findings by Endeward et al. [4,5,6] that AQP1 as well as the erythrocytic RhAG represent channels for CO_2_. In addition, they showed that the other aquaporin isoforms AQP4 and AQP5 and another member of the Rh family, the bacterial AmtB, conduct CO_2_. The same group then extended the range of aquaporin isoforms to all of the AQP isoforms 0–9 [11], and observed that CO_2_ is conducted by the AQP isoforms 0, 1, 4-M23, 5, 6, and 9, but hardly by 2, 3, 4-M1, 7 and 8. AQP0 is present in the lens fibers of the eye, and may facilitate CO_2_ release by the superficial lens-fiber cells. AQP1 is present in the RBC membrane, as discussed above, in proximal kidney tubules, where it may mediate the large amounts of CO_2_ reabsorbed in the proximal tubule [45], and almost universally in blood capillaries, where it may contribute to CO_2_ in addition to water transfer. AQP5 is strongly expressed in type I alveolar epithelial cells [46], where its CO_2_ conductivity may contribute to pulmonary CO_2_ exchange. AQP6 is present in H^+^-ATPase-carrying intracellular vesicles in renal collecting duct epithelia [47], where its CO_2_ pathway may serve the exit from the vesicle of CO_2_ produced by the intravesicular reaction H^+^ + HCO_3_^−^ → CO_2_ + H_2_O. AQP9 is present in the plasma membrane of hepatocytes [48], where its CO_2_ conductivity may be involved in providing the substrate CO_2_ to urea synthesis. It may be noted that the mitochondrial AQP8 is not a CO_2_ channel and has been shown not to contribute to the high CO_2_ permeability of the inner mitochondrial membrane [19]. 

In addition to this substantial amount of evidence derived from studies of red blood cells and gas-channel-expressing oocytes, further compelling evidence has been reported for the existence of gas channels in membranes. Some of this evidence comes from plant aquaporins, the largest group being called PIP (plasma membrane intrinsic protein). For the case of the tobacco aquaporin NtAQP1, Uehlein et al. [49] have provided the same kind of evidence that Nakhoul et al. [43] have given for human AQP1: expression in oocytes accelerated the kinetics of CO_2_ influx, as observed by intracellular pH measurement. Moreover, overexpression of NtAQP1 in tobacco plant was shown to raise membrane CO_2_ permeability and to increase plant growth. Later on, the same group confirmed this by presenting a most striking further piece of evidence for the CO_2_ channel properties of plant aquaporins [50]. They used virtually CO_2_-impermeable planar triblock-copolymer (ABA1) membranes that separated a fluid chamber with high CO_2_ partial pressure from one containing no CO_2_. The flux of CO_2_ across this layer was observed by surface pH measurement on the low CO_2_ side, using the experimental principle of Missner et al. [24]. In the absence of plant aquaporins, the membrane conducted no CO_2_ at all. After incorporation of one of the plant aquaporins NtAQP1 and NtPIP2;1, a most impressive flux of CO_2_ through the layer was observed, providing quite direct proof for the properties of these aquaporins as CO_2_ channels. Similarly, a little later this same group [23] reported measurements on lipid bilayers of l-a-Phosphatidylcholine (PC) using again the same surface pH technique. They first showed that incorporation of stigmasterol (Stig) into the bilayer drastically reduced its P_CO2_, similar to what has above been demonstrated for cholesterol. They went on to show that incorporation of NtAQP1 into the PC:Stig membrane caused an approximately 3-fold increase in P_CO2_. Lastly, they also illustrated the relevance of the background permeability for the visibility of the CO_2_ channel property of aquaporin by incorporating NtAQP1 into a pure PC membrane. The membrane without Stig had a substantially higher P_CO2_, rendering the effect of NtAQP1 almost invisible. This latter observation provides further reinforcement of the concept that gas channels are effective in membranes with a low intrinsic CO_2_ permeability, but not in membranes of high basal P_CO2_ [3,51]. 

In fishes, interesting evidence has been obtained on Rh and AQP gas channels. Perry et al. [52] have shown that the Rh proteins Rhcg1 and Rhbg show competition between the transport of CO_2_ and NH_3_, i.e., they facilitate transport of both gases: (a) in Zebrafish larvae knockdown of Rhcg1 and/or Rhbg decreased both CO_2_ and ammonia efflux across the skin of the larvae, (b) in adult zebrafish the increased efflux of CO_2_ across the gills after exposure of the animals to hypercapnia was accompanied by a decrease in ammonia efflux. The conclusion was that the Rh proteins are channels for both CO_2_ and NH_3_, which agrees with the competition between CO_2_ and NH_3_, as demonstrated by Endeward et al. [6] for RhAG of the human red cell membrane. Later, the same group [53] showed that the zebrafish aquaporin-1a1 exhibits an analogous dual role as gas channel for both CO_2_ and NH_3_. Morpholino knockdown of AQP-1a1 protein expression in zebrafish larvae, while not affecting oxygen uptake of the larvae, reduced their CO_2_ release as well as their ammonia efflux, in addition to reducing their exchange of water. Talbot et al. [53] suggest that the localization of AQP-1a1 in the yolk sac epithelium rather than its localization in RBCs is the crucial one for the transport of the two gases in the early stages of zebrafish development. 

The evidence presented in this section collectively indicates compellingly that protein CO_2_ channels exist and that they are functionally important in some cells, although not in others. 

## 7. Physiological Role of CO_2_ Channels in Mammals

As just discussed, CO_2_ channels in plants appear to have an important functional role. Uptake of CO_2_ by plants occurs, due to the low atmospheric CO_2_ partial pressure (pCO_2_) of 0.3 mmHg, along an extremely small pCO_2_ gradient. This renders membrane CO_2_ permeability crucial, especially when compared to mammals, which have an arterial pCO_2_ of 40 mmHg. Plant aquaporin-mediated CO_2_ transport has been implicated in several plant functions and importantly in plant growth [49,54,55,56]. Similarly, Perry and Gilmour and colleagues [52,53] have shown that the central function of the gills of zebrafish, or skin in larvae, to excrete ammonia and CO_2_ is greatly supported by CO_2_/NH_3_ channels. 

The contributions of CO_2_ channels of mammalian origin to CO_2_ transport have been well documented in artificial systems such as liposomes and oocytes. The only mammalian in vivo system, in which the presence, as well as the role in membrane CO_2_ permeation, have experimentally been documented thoroughly, are human red blood cells. This has already been discussed above, and will be elaborated further below. In addition, there are preliminary data showing a similar role of the AQP1 CO_2_ channel in the proximal tubular epithelium of the kidney, as will also be discussed below. 

What is the role of the RBC CO_2_ channels for CO_2_ exchange of the body? Endeward et al. [4,6] have used a mathematical model taking into account the n vivo RBC geometry in the lung capillary [57], transmembrane CO_2_ diffusion, and intraerythrocytic reaction and diffusion of CO_2_/HCO_3_^−^, to estimate the influence of P_CO2_ on the kinetics of release of CO_2_ as it occurs in the lung. Bicarbonate movements across the RBC membrane were not taken into account in this model. From these calculations, Table 2 shows that in the case of zero diffusion resistance of the RBC membrane, 50 ms are required for the red cell to release 95% of the CO_2_ that is due to be exchanged in the lung. This compares with a capillary transit time in the lung of 700 ms at rest and about 350 ms under high workload. With the diffusion resistance exerted by the normal RBC CO_2_ permeability of 0.15 cm/s, this time rises to 110 ms, still sufficiently fast under all of the conditions. Even when only one of the CO_2_ channels, either AQP1 or RhAG, is lacking, the t_95%_ of 180 ms is still short enough. However, when the RBC CO_2_ permeability falls to its basal level in the absence of both channels and assumes a value of ~0.01 cm/s, the t_95%_ increases to 1000 ms, which is critical under conditions of rest but clearly too long under heavy exercise. This predicts that a complete lack of CO_2_ channels in the RBC membrane should become apparent under conditions of maximally increased oxygen consumption. 

We have started to study whether this can be demonstrated experimentally in mice. When oxygen consumption of mice was increased to its maximum by means of the Helox technique, we found indeed a reduction of maximal oxygen consumption by 16% in mice lacking AQP1 [58]. This observation indicates a new phenotype of AQP1-kockout mice. We then further analysed the possible cause of this interesting finding, and obtained a surprising result [59]. In this second study, our group observed that the hearts of AQP1-deficient mice possess a 12% thinner wall of the left ventricle than WT mice, a 10% reduced capillary density, and a left ventricular weight normalized to tibia length reduced by 20% for males and by 8% for females. The latter finding has also been reported previously by Montiel et al. [60]. The conclusion from these results must be that, with less ventricle mass und thinner ventricle walls, the AQP1-deficient hearts are expected to exhibit a smaller maximal cardiac output and thus a lower maximal oxygen consumption. A possible cause for the reduced muscle mass and capillarisation of the AQP1-deficient hearts has been proposed by Al-Samir et al. [59] to be the known important role of AQP1 in capillary growth. Thus, the reduction of maximal oxygen consumption in AQP1-knockout mice cannot safely be attributed to the gas channel function of AQP1 in red cells or other tissues. This is not unexpected in view of the calculations of Endeward et al. [4,6] as described in Table 2 above, whose result is that in human RBCs lack of only one type of gas channel will not noticeably affect the efficiency of CO_2_ exchange in the body. This in line with the other findings of Al-Samir et al. [58] that neither Rhag-KO nor AQP9-KO (AQP9 being another CO_2_-conducting AQP of the mouse red cell membrane) show a significant reduction of maximal oxygen consumption when compared to controls. However, their finding that also Rhag-AQP1-double KO mice show no reduction of maximal O_2_ consumption beyond that already seen in AQP1-single KO mice, is difficult to reconcile with the results of Table 2. An explanation may come from unpublished work of our lab (Endeward and Gros), which shows that the CO_2_ permeability of the red cell membranes of all these gas channel-knockout mice, single as well as double, is affected to a much lesser extent by the knockouts than that of human red cell membranes by the lack of one, either AQP1 or RhAG. Our conclusion at present is therefore that mouse red cells deficient in these gas channels are not a suitable model to study the significance of RBC gas channels for the human organism; their contribution to the CO_2_ permeability of the red cell membrane of mice appears to be too small. New approaches are required to assess the role of AQP1 in gas exchange in humans. Likewise, it will be important to study the contribution to CO_2_ exchange in the body of other CO_2_-conducting AQP isoforms such as the AQP5 of the lung. 

Another physiological process that may strongly depend on protein CO_2_ channels, is the bicarbonate reabsorption of the proximal kidney tubule. It is quite conceivable that the epithelial cell membrane adapts to this situation by establishing a high membrane CO_2_ permeability, which has, however, not been quantitated so far. The apical membrane of kidney proximal tubules has a fraction of cholesterol per total membrane lipids of about 50% [60], which would suggest a rather low intrinsic CO_2_ permeability for this membrane (see the above discussion). This permeability may be increased substantially by the high level of aquaporin 1 expressed in this membrane [61]. Indeed, Boron [45] has reported preliminary evidence showing that about 60% of proximal HCO_3_^−^ reabsorption is lost, when AQP1 is missing in the kidney, indicating that transepithelial CO_2_ flux is reduced by 60% in the absence of AQP1. 

## 8. Conclusions

We believe there is now ample and largely undisputed evidence that protein CO_2_ channels exist in several mammalian and non-mammalian membranes. We have presented here, in addition, more recent evidence indicating that membrane CO_2_ permeabilities can be modulated over a wide range by CO_2_ channels, and probably even more often by the cholesterol content of the membrane. One physiological purpose of this modulation seems to be to adapt the ease of CO_2_ permeation across the membrane to the amount of CO_2_ produced per time by the specific cell. This becomes apparent from an excellent linear relation between cellular rates of oxygen consumption and membrane CO_2_ permeabilities. In special cases, membrane CO_2_ permeabilities are increased above the level resulting from membrane cholesterol by the incorporation of CO_2_ channels, such as aquaporin or Rhesus protein. Examples are the red blood cells, whose CO_2_ permeability is 10x the value expected from their membrane cholesterol content. Their high CO_2_ permeability most certainly is related to the specialized role of red cells in CO_2_ exchange in the body. Another example may be the apical membrane of proximal tubules, where AQP1 may serve to accomplish the high transmembrane CO_2_ fluxes necessary for proximal bicarbonate reabsorption. Because of species-specific properties of mice that differ from those of humans, it has so far not been possible to use this animal model to investigate the role of protein gas channels in systemic CO_2_ (or O_2_) exchange. Further efforts are necessary to evaluate this role. 

## Figures and Tables

**Table 1:** CO_2_ permeabilities of cells and organelles of various mammalian tissues.

Type of Cell/Organelle	P_CO2_ (cm/s)	
Apical membrane PCE ^a^	0.0015	Endeward and Gros [8]
MDCK ^b^	0.017	Itel et al. [3]
Basolateral membrane PCE ^a^	0.022	Endeward and Gros [8]
Rat Cardiomyocytes	0.1	Arias-Hidalgo et al. [18]
Human Erythrocytes	0.15	Endeward et al. [4,6]
Mitochondria ^c^	0.33	Arias-Hidalgo et al. [19]

^a^ proximal colon epithelium of guinea pig; ^b^ Madin Darby canine kidney cells; ^c^ isolated from rat liver.

**Table 2:** Time required by human red cells (RBCs) to complete CO_2_ release during passage through the lung capillary by 95% (t_95%_).

	P_CO2_ (cm/s)	t_95%_ (ms)
No membrane resistance	∞	50
Normal membrane permeability	0.15	110
AQP-1_null_ or Rh_null_ RBCs	0.07	180
Basal permeability (AQP-1_null_ + DIDS)	0.01	1000

Values calculated for different CO_2_ permeabilities of the cell membrane. Intraerythrocytic carbonic anhydrase activity is assumed to be nonlimiting (from: Endeward et al. [6]). On the basis of the in vivo shape of red cells in the lung capillary [57], RBCs were approximated in the model by a sphere of radius 2.5 μm.
